# Chloride Binding Behavior and Pore Structure Characteristics of Low-Calcium High-Strength Cement Pastes

**DOI:** 10.3390/ma17133129

**Published:** 2024-06-26

**Authors:** Ziwei Wang, Minglei Guo, Chunlin Liu, Zhong Lv, Tengfei Xiang, Shunquan Zhang, Depeng Chen

**Affiliations:** School of Civil Engineering and Architecture, Anhui University of Technology, Ma’anshan 243002, China; wzw1300943383@163.com (Z.W.); mingleiguo@ahut.edu.cn (M.G.); zhonglv1982@163.com (Z.L.); xiangtf@ahut.edu.cn (T.X.); sqzhang@ahut.edu.cn (S.Z.)

**Keywords:** low-calcium high-strength cement, chloride binding, mineral admixtures, pore structure

## Abstract

While Portland cement produces large amounts of carbon dioxide, low-calcium high-strength cements effectively reduce carbon emissions by decreasing the proportion of high-calcium minerals. In order to enhance the practical application value of low-calcium high-strength cement, the effects of mineral admixtures on the chloride binding capacity and pore structure characteristics of low-calcium high-strength cement pastes were investigated by equilibrium method and mercury intrusion method. The results showed that the chloride binding capacity of low-calcium high-strength cement pastes is superior to that of Portland cement. Fly ash and slag enhance this capacity by promoting monosulfoaluminate and C-S-H gel formation, with fly ash being more effective. Ground limestone also boosts chloride binding when incorporated at less than 10 wt%. However, sulfates have a more significant negative impact on chloride binding capacity in low-calcium high-strength cement pastes compared to Portland cement. The porosity of low-calcium high-strength cement pastes exhibits contrasting trends with the addition of fly ash, ground limestone, and slag. Fly ash and limestone initially coarsen the pore structure but later facilitate the transition of larger pores to smaller ones. In contrast, slag initially has little impact but later promotes the conversion of large capillary pores to medium ones, optimizing the pore structure. Notably, above 10 wt% fly ash, the critical pore diameter decreases with additional fly ash except at 10% where it increases for 3 days. Ground limestone enlarges the critical pore diameter, and this effect intensifies with higher content. During early hydration, slag decreases the critical pore diameter, but its impact diminishes in later stages.

## 1. Introduction

Since the signing of the “Kyoto Protocol”, countries around the world have reached a consensus on reducing greenhouse gas emissions to cope with global warming. Europe, the United States, Japan, and other countries have proposed to achieve the goal of “Carbon Neutrality” by 2050. As the largest developing country in the world, China made a commitment at the 75th United Nations General Assembly that it would strive to achieve the “peak of carbon dioxide emissions” by 2030 and achieve “carbon neutrality” by 2060. As one of the major sources of CO_2_ emission in China, the CO_2_ emission of the cement industry in 2020 is up to 1.23 billion tons, and its CO_2_ emission accounts for 14.3% of the total CO_2_ emission in China, therefore, the cement industry is an important field for realizing “carbon neutrality “ in China [1]. The CO_2_ emissions from the cement industry mainly come from two aspects: one is the CO_2_ released by the high-temperature decomposition of limestone during the clinker burning process (about 60%); the other is the CO_2_ emissions from fuel combustion during the clinker burning process (about 40%) [2]. The main mineral of the traditional silicate clinker is calcium silicate (3CaO·SiO_2_, C_3_S). The calcium silicate has a high firing temperature (about 1450 °C) and the relative content of CaO is also higher than that of dicalcium silicate (2CaO·SiO_2_, C_2_S). Therefore, the energy consumption and carbon emission of the C_3_S firing process are relatively higher [3]. In general, the high carbon emission of conventional silicate cement is due to its high calcium mineral design. On the basis of reducing energy consumption, reducing carbon emission, and replacing silicate cement for mass production and application, the national key research and development “key preparation technology and demonstration application of low-environmental-load high-performance cementing materials” has developed low-calcium high-strength cement. The novel low-calcium cement has the advantages of low energy consumption, low carbon emission, high strength, wide application range, etc.

The durability of reinforced concrete structures has been a hot research issue. In harsh environments, the durability of reinforced concrete structures is subjected to ionic erosion, carbonization, freeze–thaw cycle, and load stress [4], resulting in structural durability reduction and huge economic loss. The failure of the passivation film on the steel surface caused by chloride ion erosion is the main reason for structural instability [5]. The chloride ion curing capacity of cement-based materials, also known as chloride ion curing capacity, has a great influence on the diffusion of chloride ions in cement-based materials [6,7,8]. In order to save costs and improve performance, mineral admixtures are usually used as auxiliary cementing materials. Many scholars have studied the effect of mineral admixtures on the curing ability of chloride ions of cement-based materials. The addition of fly ash can improve the chloride binding capacity of cement, because fly ash contains a large amount of Al_2_O_3_, which will accelerate the formation of chloride ion binding products Fs (Friedel’s salt) [9,10] and Ks (Kuzel’s salt) [11] and increase the chemical binding capacity. With the increase in fly ash content, the chloride ion binding capacity of cement first increases and then decreases [12], and the study by Shi et al. [13] shows that the optimal fly ash content is 25%. Similar to fly ash, with the increase in slag content, the chlorine ion binding capacity of cement also shows a trend of first increasing and then decreasing, but the optimal content is controversial [14,15]. With the increase in ground limestone content, the Fs content increases, and the binding capacity of chloride ion is gradually enhanced with the increase in ground limestone content and increases first and then decreases with the increase in ground limestone fineness [16]. In practical engineering, the erosion of chloride ions is often accompanied by the erosion of sulfate ions, sodium ions, magnesium ions, and other ions, among which sulfate ions have a great impact on the transmission of chloride ions in concrete [17]. SO_4_^2−^ mainly affects the stability of Fs and Ca/Si of C-S-H gel to change the binding capacity of chloride ions [18], but there is an interaction between Cl^−^ and SO_4_^2−^. Since the transmission rate of Cl^−^ in cement-based materials is higher than that of SO_4_^2−^, the generated Fs will fill the pores and inhibit SO_4_^2−^ from entering concrete [19]. In addition, the pore structure characteristics also significantly affect chloride ion transport in cement-based materials. Li [20] et al. believe that the porosity and pore diameter distribution are the main factors affecting the variation of chloride ion diffusion coefficient in concrete; Zeng [21] et al. have shown that the connectivity of pores can also significantly affect the chloride ion penetration resistance of cement-based materials.

There are many studies on chloride binding capacity and pore structure characteristics of ordinary Portland cement-based materials, low-calcium high-strength cement has a different mineral composition from Portland silicate cement, which will significantly affect the transport mechanism of chloride ions. The research on the chloride binding behavior and pore structure characteristics of low-calcium high-strength cement can provide a theoretical basis for practical application and has great significance for reducing carbon emissions in the cement industry. In this paper, the effect of mineral admixture and sulfate on the chloride binding capacity of low-calcium high-strength cement pastes was studied by equilibrium method [22], and the pore structure characteristics of low-calcium high-strength cement pastes were studied by mercury intrusion method.

## 2. Materials and Methods

### 2.1. Materials

The low-calcium high-strength cement used in the test is one of the research achievements of the key research and development project “key preparation technology and demonstration application of low environmental-load high-performance cementing material”, which is recorded as LC; the cement used in the control group is P·I 42.5 silicate cement, which is recorded as C; the fly ash is grade I fly ash, which is recorded as FA (fly ash); the ground limestone used in the test is produced by Henan Borun New Materials Co., Ltd. (Xinxiang, China), and is recorded as GL (ground limestone); the slag is grade S95, and is recorded as MP; The chemical composition is provided by the manufacturer, while the mineral composition is derived from XRD analysis. See Section 2.3.1 for XRD analysis methods. The chemical compositions of LC, C, FA, GL, and MP are shown in Table 1, and the mineral compositions of LC and C are shown in Table 2; NaCl and Na_2_SO_4_ were analytically pure. In the test, deionized water is used for specimen preparation and chemical analysis.

### 2.2. Preparation of Sample

The 40 mm × 40 mm × 40 mm cubic low-calcium high-strength cement paste test specimens were formed [22], and the mix proportions are shown in Table 3. The specimen was placed in saturated lime water at (20 ± 2) °C for maintenance for 56 d.

The 40 mm × 40 mm × 40 mm cube of low-calcium high-strength cement–mineral admixture composite paste test specimen was formed. The mixture ratio is shown in Table 3. After being cured under standard conditions for 24 h, the formwork was removed, and the specimen was further cured under standard conditions for 3 days, 7 days, and 28 days. The test piece was then crushed, and 3~5 mm of cement stone fine particles were collected. These particles were immersed in absolute ethyl alcohol for 7 days, dried in a vacuum drying oven for 24 h, and placed in a sealed and dry glass bottle for mercury intrusion testing. 

### 2.3. Characterization

#### 2.3.1. XRD Analysis

X-ray diffraction (XRD) analysis of powder samples was carried out by D8ADVANCE X-ray diffractometer produced by Germany Bruker Company. The main parameters of the equipment are as follows: copper target; power: 3 KW; scanning mode of goniometer: θ/θ mode; scanning range: −3°~150°; precision of goniometer: 0.0001°; accuracy of 2θ angle: ≤0.02°. The chloride ion concentration in the solution is determined by potentiometric titration.

#### 2.3.2. Mercury Intrusion Analysis 

The samples were analyzed by AutoPore IV 9500 full-automatic mercury porosimeter made by Micromeritics Instrument Corporation, Norcross, GA, USA. The pressure range was 0~227.51 MPa and the corresponding pore diameter was 0.005~120 μm.

#### 2.3.3. The Test of Chloride Binding Capacity

The test specimen was crushed and ground and cement stone particles with a particle size of 0.25 mm–2.0 mm were collected. These particles were then dried in a vacuum drying oven at 30 °C for 24 h and placed in a sealed and dry glass bottle for the chloride binding test. A 5 g amount of the prepared sample granules was weighed, and placed into a conical flask, and 50 mL of each of the 4 different types of chlorine salt solution were added. The composition of the chloride salt solution is shown in Table 4. Seal the bottle with plastic wrap to prevent evaporation. During the experiment, the ambient temperature is kept at 20 ± 2 °C, and the particle sample is shaken at certain times to ensure that the particle sample is in full contact with the solution; after the sample particles are soaked for 35 d, the suspension is subjected to solid–liquid separation by using a funnel, and the obtained solution is used for determining the chloride ion concentration, and the hydration of the solid is stopped by absolute ethyl alcohol, and the sample to be measured is prepared by grinding powder after vacuum drying for XRD analysis. The chloride ion concentration in the solution is determined by potentiometric titration. The precision of the titration volume is 0.0001 mL, indicating a high degree of accuracy in measuring minute changes in volume down to the fourth decimal place, or one-tenth of a microliter. The titration standard solution is 0.1 mol/L AgNO_3_. Before the solution titration, the following treatment shall be carried out: 3 mL of the solution to be tested was placed in a beaker with a pipettor, and 100 mL of deionized water and 5 mL of 2 mol/L HNO_3_ solution was added at the same time. Each solution sample is titrated for three times, and the average value is taken. The calculation of chloride ion curing capacity is referred to in the literature [22], and calculation formula for curing amount of chloride ion is as follows:

$$C_{b}=\frac{35.45V(C_{0}-C_{1})}{W}$$

where *C_b_* is the amount of chloride ion curing per unit mass of slurry, mg/g; *V* is the volume of the immersion solution, *V* = 50 mL; *C*_0_ and *C_i_* are, respectively, the chloride ion concentration after initial and equilibrium of the solution, mol/L; *W* is the mass of sample granule added during the equilibrium test, *W* = 5 g;

## 3. Results

### 3.1. Chloride Binding Capacity

#### 3.1.1. Chloride Binding Capacity of Low-Calcium High-Strength Cement Pastes

The data in Table 5 display the bound chloride content of both low-calcium high-strength cement pastes and Portland cement pastes after achieving equilibrium in a chloride salt solution. The following can be derived from the data: under the condition of the same chloride ion concentration, the chloride binding capacity of the low-calcium high-strength cement pastes exceeds that of the Portland cement pastes by 10.6%, thereby suggesting that the chloride binding capacity of the low-calcium high-strength cement pastes is superior to that of the Portland cement pastes. 

**Figure 1:**
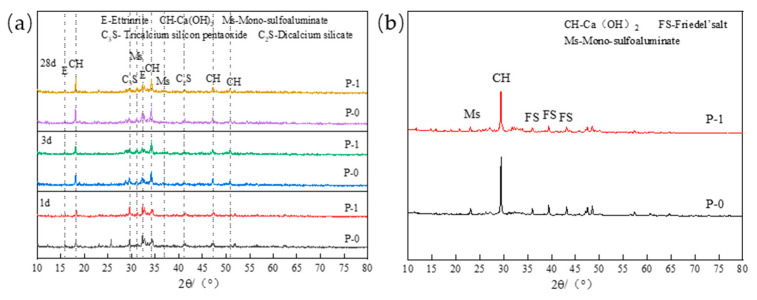
(**a**) P-0 and P-1 hydration productions at each age before immersion; (**b**) P-0 and P-1 hydration productions after immersion.

Figure 1a shows XRD patterns of hydration productions at 1 d, 3 d, and 28 d before immersion of P-0 and P-1. It can be seen from the figure that in the early stage of hydration, the Ms (SO_4_^2−^-AFm) generated by low-calcium high-strength cement is higher than that of Portland cement, both of which contain a large amount of unhydrated C_3_S and C_2_S. With the progress of hydration, the diffraction peaks of both C_3_S and C_2_S decrease. In the later stage of hydration, the Ca(OH)_2_ diffraction peaks of both cement and P-0 increased, and the intensity of Ca(OH)_2_ diffraction peak in P-0 was greater than that of P-1, indicating that more C-S-H gel was formed in the later stage of low-calcium high-strength cement pastes. Figure 1b is an XRD pattern of the hydrated product after immersion. It can be seen from the figure that the content of Fs in P-0 is higher than that of P-1, and Fs is the product of the chemical bound of Cl^−^ by AFm compounds. Therefore, the chloride binding capacity of low-calcium high-strength cement pastes is stronger than that of Portland cement pastes.

#### 3.1.2. Effect of Different Mineral Admixtures on the Chloride Binding Capacity of Low-Calcium High-Strength Cement Pastes

**Figure 2:**
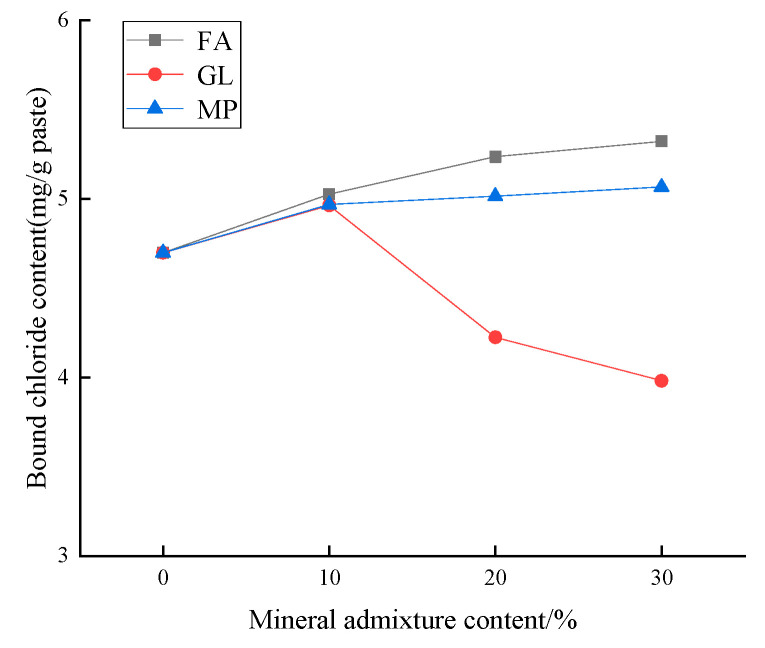
Effect of different mineral admixtures on chloride binding capacity of low-calcium high-strength cement pastes. (Error: 1.3–3.6%).

Figure 2 shows the effect of different mineral admixtures on the chloride binding capacity of low-calcium high-strength cement pastes. It can be seen from the figure that the use of fly ash and slag can improve the chloride binding capacity of the low-calcium high-strength cement pastes, and the content of ground limestone greater than 10% will reduce the chloride binding capacity of the low-calcium high-strength cement pastes. With the increase in the content of fly ash and slag, the content of bound chloride in the low-calcium high-strength cement pastes gradually increases. The content of bound chloride ions in P-0, P-FA10, P-FA20, and P-FA30 is 4.70, 5.03, 5.24, and 5.32 mg/g, respectively. When the content of fly ash increases by 10%, the content of bound chloride ions in the low-calcium high-strength cement pastes is increased by 7.0~13.2% compared with that of the P-0 group. However, the effect of fly ash on the chloride binding capacity of low-calcium high-strength cement pastes is less with the increase in the content of fly ash. The content of bound chloride in P-MP10, P-MP20, and P-MP30 was 4.97, 5.02, and 5.07 mg/g, respectively, when slag content increased by 10%, the content of bound chloride ions in composite cement pastes was 5.7~7.9% higher than that of P-0 group, as the amount of GL increases, the content of bound chloride ions in the low-calcium high-strength cement pastes is gradually reduced. P-GL10 P-GL20 and P-GL30 content of bound chloride were, respectively, 4.96, 4.22, and 3.98 mg/g, the content of bound chloride ions in the composite cement pastes increased at first and then decreased with the increase in the content of ground limestone, compared with the P-0 group, the content of bound chloride ions in the composite cement pastes with 10% of ground limestone is increased by 5.5%. The content of bound chloride in 30% ground limestone composite cement paste decreased by 15.3% compared with the P-0 group. Thus, at the same dosage, the addition of fly ash and slag can more effectively improve the chloride binding capacity of the low-calcium high-strength cement pastes, fly ash is more effective.

**Figure 3:**
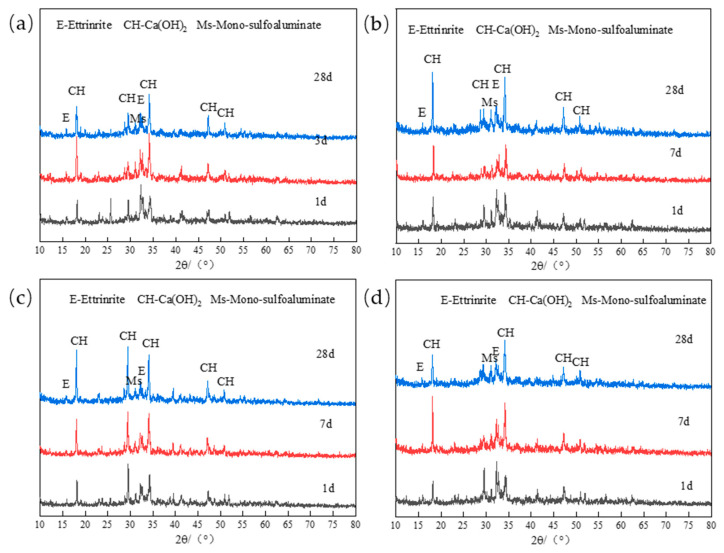
(**a**) P-0 hydration products of each age; (**b**) FA10 hydration products of each age; (**c**) GL10 hydration productions at each age; (**d**) MP10 hydration productions at each age.

The chloride binding capacity of cement pastes is mainly related to its hydration product AFm compound and C-S-H gel [21]. AFm group compounds bind chloride ions by chemically reacting with Cl^−^ to form Fs. The content of AFm generated by hydration of low-calcium high-strength cement will significantly affect its chloride binding capacity; C-S-H gel binds chloride ions through physical adsorption of Cl^−^. However, since most C-S-H gels are amorphous, and their production amount is proportional to the production of Ca(OH)_2_, Ca(OH)_2_ can be used to characterize the physical adsorption of Cl^−^ by C-S-H gels [9]. In order to analyze the influence of mineral admixtures on the hydration productions of low-calcium high-strength cement, Figure 3 compares the hydration productions of low-calcium high-strength cement pastes in 1 d, 3 d, and 28 d. From the analysis of Figure 3a–d, it can be seen that the formation of AFt is mainly in the early stage of hydration. With the progress of hydration, the content of AFt decreases continuously, while the content of Ms(SO_4_^2−^-AFm) increases with the increase in hydration age. By analyzing the diffraction peak intensity of Ca(OH)_2_, it can be seen that the yield of C-S-H gel increases with the increase in hydration age, and the variation is relatively obvious from 7 to 28 d, which is due to the low hydration activity of C_2_S in low-calcium high-strength cement, and will slowly hydrate to form C-S-H gel within 7–28 d [23,24].

**Figure 4:**
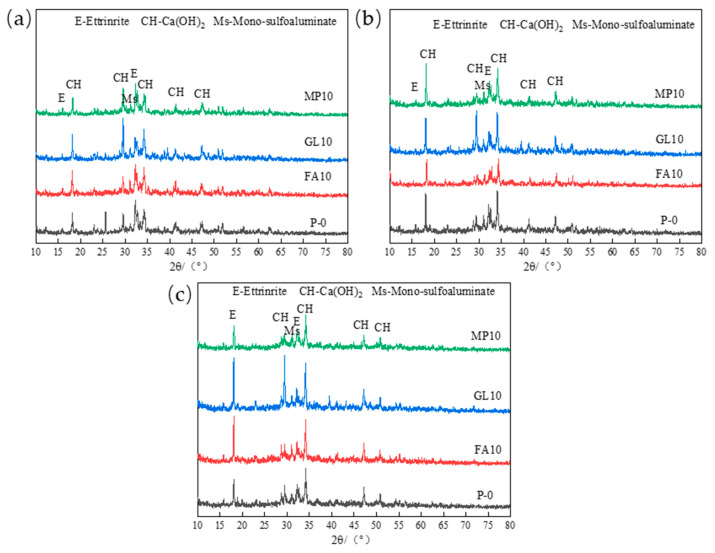
Effect of mineral admixtures at different ages on the hydration productions of low-calcium high-strength cement: (**a**) 1 d hydration productions; (**b**) 3 d hydration productions; and (**c**) 28 d hydration product.

Figure 4 shows the XRD patterns of hydration productions of low-calcium high-strength cement pastes at different ages under 10% fly ash, ground limestone, and slag. By analyzing Figure 4a,b, it can be seen that the addition of 10% fly ash has little effect on the formation of AFt and C-S-H in 1~3 d of low-calcium high-strength cement pastes, but it will significantly improve the formation of 28 d Ms of low-calcium high-strength cement pastes. Yang Z et al. study [25] shows that the yield of AFm phase in cement hydration productions is mainly related to the content of Al_2_O_3_. The higher the content of Al_2_O_3_ available for bonding, the more content the AFm phase is generated, and a large amount of Al_2_O_3_ is contained in fly ash, which is beneficial to the formation of the AFm phase in the later stage of cement hydration. The incorporation of 10% ground limestone can improve the production of AFt and Ca(OH)_2_ at each age, in one aspect, a large amount of CaO in the ground limestone can be hydrated to generate Ca(OH)_2_, but also provides Ca element for the generation of C-S-H gel; in another aspect, the ground limestone can improve the stability of the AFt, favoring the generation of AFt [11]. The addition of 10% slag has little effect on the formation of Ms and C-S-H in low-calcium high-strength cement. 

**Figure 5:**
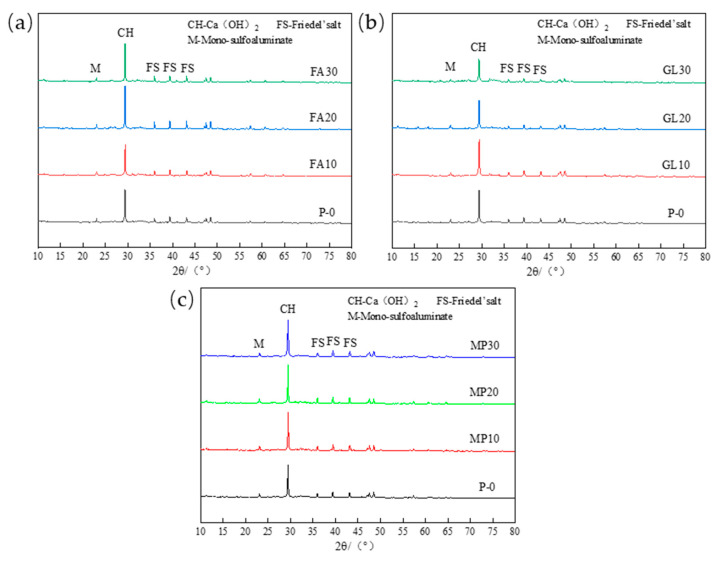
(**a**) Effect of fly ash on chloride ion corrosion products of low-calcium high-strength cement; (**b**) effect of ground limestone on chloride ion corrosion products of low-calcium high-strength cement; (**c**) effect of slag on chloride ion corrosion products of low-calcium high-strength cement.

Figure 5 shows the X-ray diffraction pattern of the hydrated pastes of low-calcium high-strength cement after immersion in the solution. It can be seen from the figure that with the increase in fly ash content, the content of Friedel’s salt (Cl^−^-AFm) and Ms (mono-sulfoaluminate) slightly increases, and the content of Ca(OH)_2_ significantly increases, which indicates that the improvement of bound chloride by fly ash for low-calcium high-strength cement pastes is mainly reflected in the physical adsorption of Cl^−^ by C-S-H gel, which is due to the pozzolanic effect of fly ash, which promotes the formation of more C-S-H gel in the later stage, thus binding more Cl^−^. The analysis of Figure 5b shows that when the content is less than 10%, the content of Ms and Fs increases with the increase in ground limestone content, and the content of Ca(OH)_2_ increases significantly; when the content is more than 10%, the content of Ms, Fs, and Ca(OH)_2_ all decrease with the increase in the content of ground limestone. It can be seen that the effect of ground limestone on the chloride binding capacity of low-calcium high-strength cement pastes is mainly reflected in physical adsorption, and too high content of ground limestone is unfavorable to the chloride binding capacity of low-calcium high-strength cement pastes, and the optimal content of ground limestone is 10%. The analysis of Figure 5c shows that the contents of Ms, Fs, and C-S-H gels all increase with the increase in slag content, but as the slag content exceeds 10%, the contents of Ms, Fs, and C-S-H gels increase less. 

#### 3.1.3. Effect of Different Concentrations of SO_4_^2−^ on the Chloride Binding Capacity of Low-Calcium High-Strength Cement Pastes

**Figure 6:**
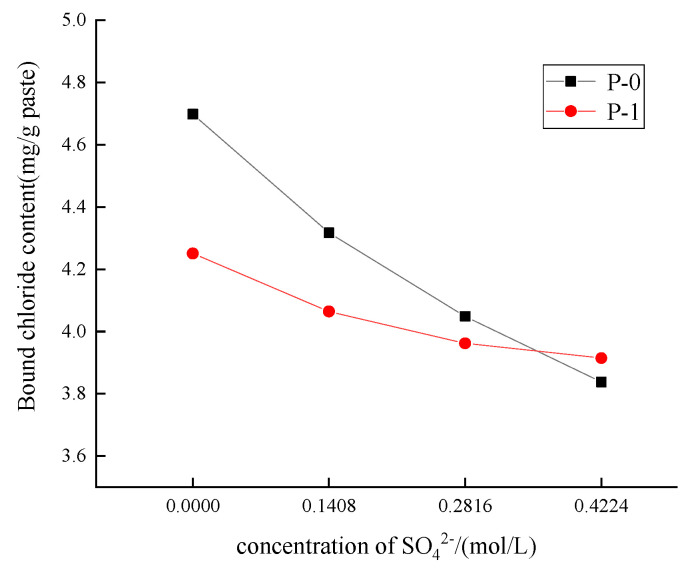
Effect of different concentrations of SO_4_^2−^ on chloride binding capacity of low-calcium high-strength cement. (Error: 2.9–4.2%).

Figure 6 shows the effect of different concentrations of SO_4_^2−^ on the chloride binding capacity of low-calcium high-strength cement pastes. It can be seen from Figure 6 that the bound chloride amount of low-calcium high-strength cement pastes and Portland cement pastes both decrease with the increase in SO_4_^2−^ concentration. With the increase in SO_4_^2−^ concentration from 0 to 0.4224 mol/L, the bound chloride in low-calcium high-strength cement pastes is reduced by 18.3%, and the bound chloride in Portland cement pastes is reduced by 8%. The bound chloride of Portland cement paste is lower than that of calcium high-strength cement pastes. This is because SO_4_^2−^ mainly changes the chloride binding capacity by influencing Friedel’s salt stability [26], that is, SO_4_^2−^ will replace Cl^−^ in Fs, decomposing the Fs salt to form AFt [27]. Because C_2_S, the C_3_S content is higher, so that the hydrated product C-S-H gel is more than the calcium high-strength cement, the chemically bound Fs content is less, low-calcium high-strength cement affected by SO_4_^2−^.

**Figure 7:**
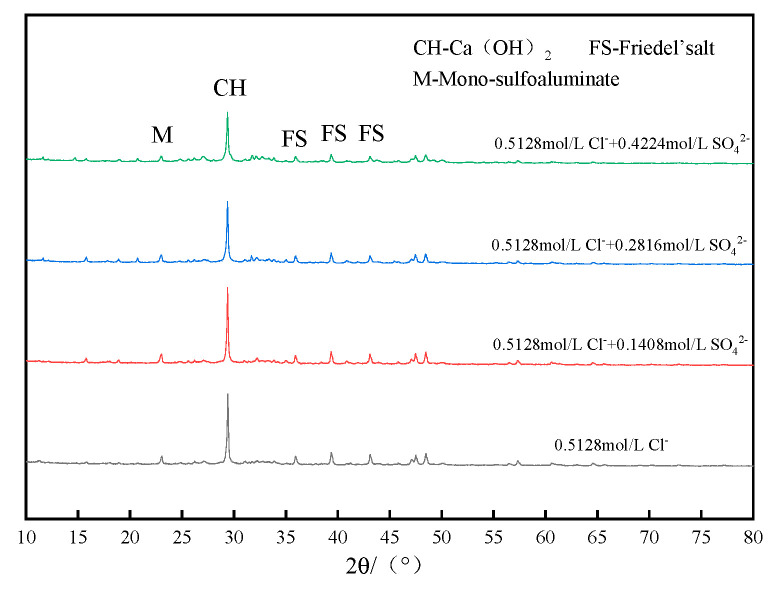
Effect of SO_4_^2−^ concentration on chloride erosion products of low-calcium high-strength cement pastes.

The effect of SO_4_^2−^ concentration on chloride ion erosion products of low-calcium high-strength cement pastes is analyzed in Figure 7, with the increase in SO_4_^2−^ concentration, the contents of Ms and Ca(OH)_2_ are continuously reduced, and the generation amount of Fs is also continuously reduced, so the increase in SO_4_^2−^ concentration reduces the chloride binding capacity of low-calcium high-strength cement pastes. 

### 3.2. Pore Structure

#### 3.2.1. Porosity

**Figure 8:**
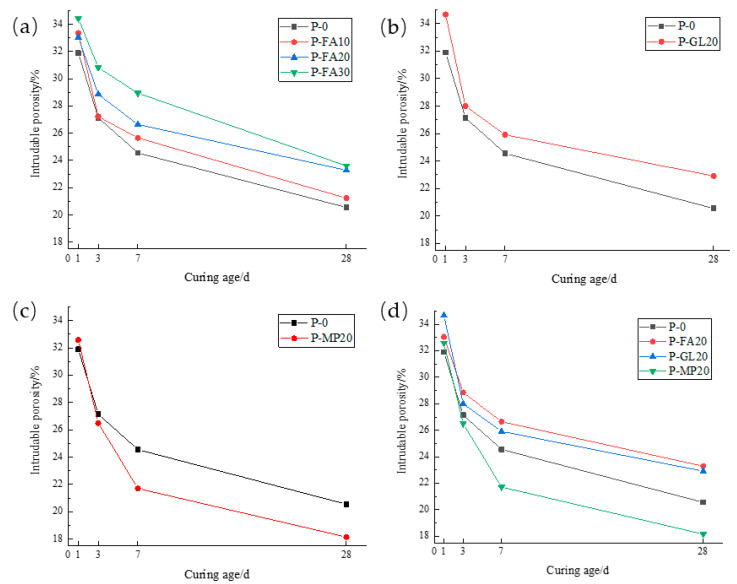
Effect of mineral admixtures on porosity of low-calcium high-strength cement pastes. (**a**) fly ash; (**b**) ground limestone; (**c**) slag; (**d**) the same amount of mineral admixture.

Figure 8a depicts how fly ash content impacts the porosity of low-calcium high-strength cement pastes. As hydration age increases, porosity decreases, sharply from 1 to 3 days and gradually from 7 to 28 days. Within each age range, higher fly ash content leads to greater porosity. Specifically, at 1 d, P-FA30 porosity rose 8.0% compared to P-0; at 3 d, it rose 13.6%; at 7 d, 17.9%; and at 28 d, 11.1%. This shows that fly ash’s impact on porosity initially rises then decreases with hydration age, peaking at 7 d. Figure 8b illustrates the impact of ground limestone content on the porosity of low-calcium high-strength cement pastes. As hydration age increases, the porosity of 20% ground limestone cement pastes decreases, sharply from 1 to 3 days and gradually from 7 to 28 days. In each age range, higher limestone content raises the porosity. Specifically, at 1 d, P-GL20 porosity rose 8.7% compared to P-0; at 3 d, it rose 3.2%; at 7 d, 5.5%; and at 28 d, 11.5%. This suggests that limestone’s effect on porosity first decreases and then increases with hydration age, with the lowest impact at 3 d. Figure 8c shows that adding 20% slag to low-calcium high-strength cement pastes decreases its porosity as the hydration age increases. Similar to fly ash and limestone, porosity drops sharply from 1 to 3 days and then more gradually from 7 to 28 days. Initially, slag slightly increases porosity, but at 7 and 28 days, it reduces porosity by 11.6% and 11.7%, respectively. Overall, slag incorporation decreases porosity in these cement pastes. Figure 8c compares the effects of 20% fly ash, ground limestone, and slag on the porosity of low-calcium high-strength cement pastes. Fly ash and limestone both increase porosity at all ages, with limestone having a more significant effect, which is undesirable for pore structure. In contrast, slag slightly increases porosity at 1 d but significantly reduces it at later ages. 

#### 3.2.2. Pore Diameter Distribution

(1) Fly ash

**Figure 9:**
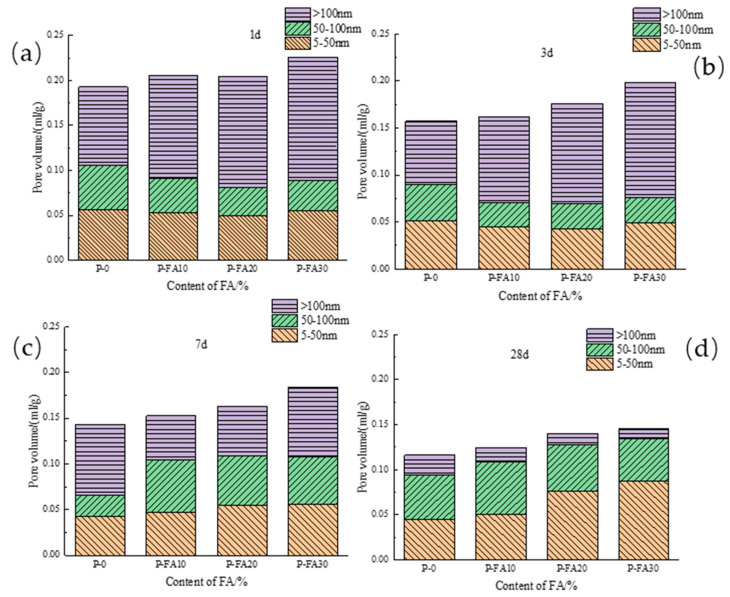
Pore diameter distribution of fly ash-mixed low-calcium high-strength cement pastes: (**a**) 1 d; (**b**) 3 d; (**c**) 7 d; and (**d**) 28 d.

Figure 9 depicts the influence of fly ash on the pore diameter distribution of low-calcium high-strength cement pastes. At 1 d and 3 d, large pores (>100 nm) increase with fly ash content, while medium pores (50–100 nm) decrease. Small pores (<50 nm) remain largely unaffected, indicating pore structure coarsening before 3 d. At 7 d, the three pore sizes are balanced. An appropriate fly ash amount transitions large pores to medium and small pores, refining the structure. However, excessive fly ash still coarsens the pore structure, with a greater coarsening effect than refinement. At 28 d, the pore diameter distribution of low-calcium high-strength cement pastes is dominated by large and small pores. Large pores decrease while medium pores first increase and then decrease with fly ash content. Small pores continuously increase. Fly ash promotes the transition from large and medium pores to small pores in later hydration stages. As hydration progresses, large pores (5–50 nm) decrease. Fly ash initially reduces AFt content, reducing pore filling and roughening the pore structure. However, over time, the C-S-H gel formed by fly ash’s pozzolanic reaction fills large and medium pores, increasing the content of small pores.

(2) Ground limestone

**Figure 10:**
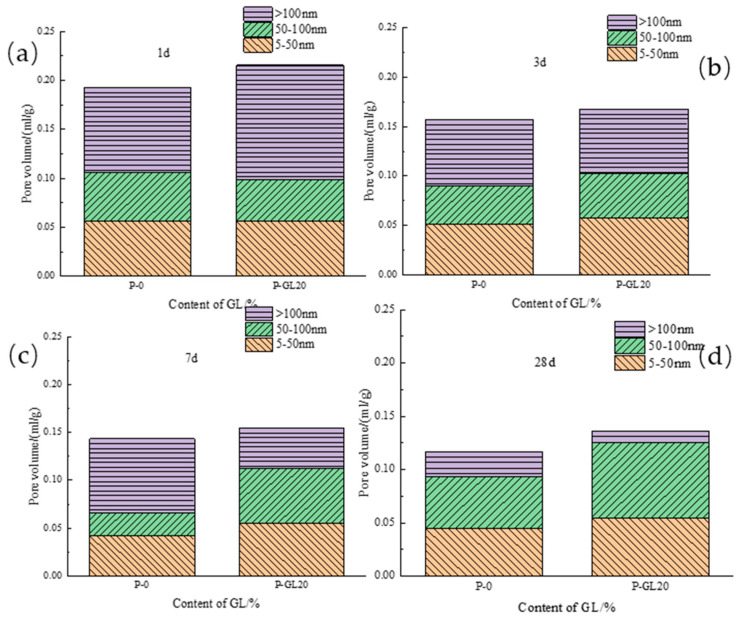
Pore diameter distribution of GL-mixed low-calcium high-strength cement pastes: (**a**) 1 d; (**b**) 3 d; (**c**) 7 d; and (**d**) 28 d.

Figure 10 demonstrates the impact of ground limestone on the pore diameter distribution of low-calcium high-strength cement paste. In 1 d, large pores increase while medium pores decrease with higher limestone content, and small pores remain unaffected. This coarsens the pore structure similar to fly ash. At 3 d, medium and small pores slightly increase. By 7 d and 28 d, the medium and small pores in cement paste with 20% limestone are significantly enlarged, indicating limestone promotes the transition from large to medium and small pores. 

(3) Slag

**Figure 11:**
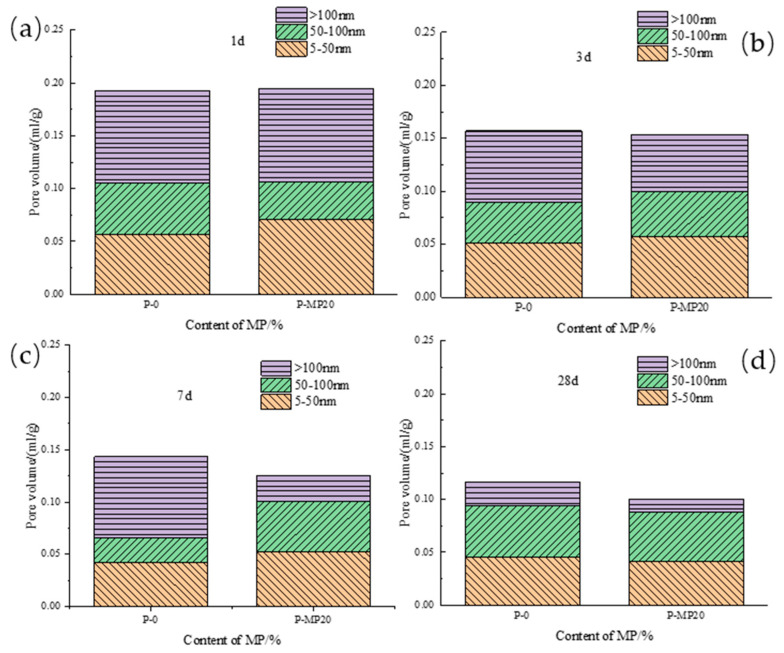
Pore diameter distribution of slag-mixed low-calcium high-strength cement pastes: (**a**) 1 d; (**b**) 3 d; (**c**) 7 d; and (**d**) 28 d.

Figure 11 illustrates the effect of slag on pore diameter distribution in low-calcium high-strength cement pastes. In 1 d, large pores remain stable, while small pores slightly increase with slag content. At 7 d, large pores significantly decrease, and medium pores increase, indicating slag promotes the transition from large to medium pores. By 28 d, cement paste with 20% slag has reduced large pores, while medium and small pores remain largely unchanged.

#### 3.2.3. Critical Pore Diameter

(1) Fly ash

**Figure 12:**
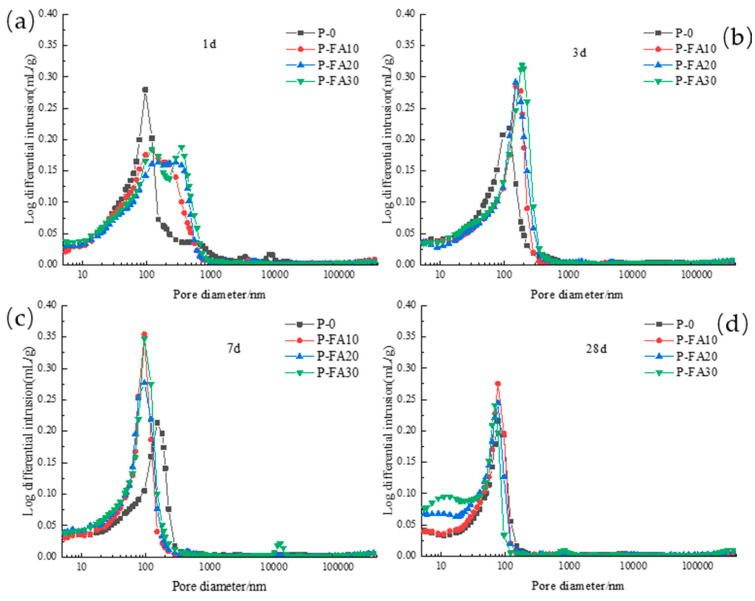
Differential curve of pore diameter distribution of low-calcium high-strength cement pastes with different dosages of fly ash: (**a**) 1 d; (**b**) 3 d; (**c**) 7 d; and (**d**) 28 d.

Figure 12 shows the influence of fly ash on the pore diameter distribution differential curve of low-calcium high-strength cement pastes at different hydration ages. It can be found that at the age of 1 d, with the increase in fly ash content, the peak moves to the direction of large pore diameter, indicating that the addition of fly ash coarsens the pore structure of low-calcium high-strength cement pastes; at the age of 3 d, there is only one peak in each group, and with the increase in fly ash content, the peak moves to the direction of large pore diameter, and the peak intensity also increases with the increase in fly ash content, indicating that the addition of fly ash increases the macropore content of low-calcium high-strength cement; at the age of 7 d, the addition of fly ash causes the peak to move to the direction of small pore diameter and the peak intensity is greatly improved, fly ash promoted the transformation from macropore to microporosity at the age of 28 d, the P-FA20 and P-FA30 groups again showed round peaks, and the peak intensity is increased along with the increase in the fly ash doping amount, it shows that fly ash refines the pore structure of low-calcium high-strength cement pastes.

**Figure 13:**
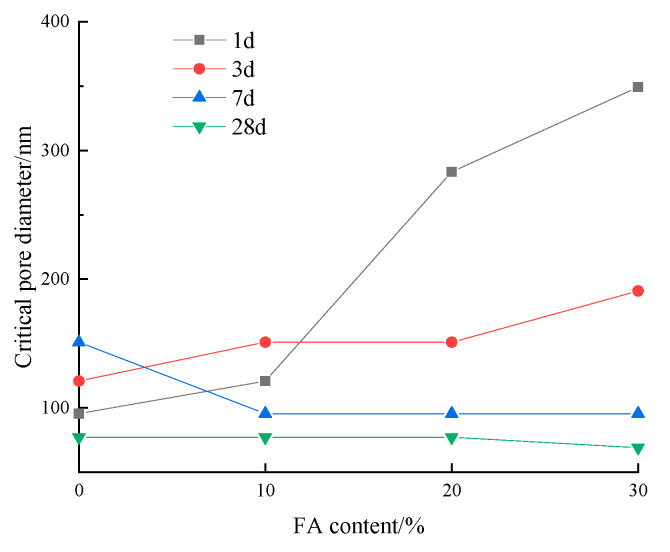
Effect of fly ash content on the critical pore diameter of low-calcium high-strength cement pastes.

Figure 13 demonstrates the influence of fly ash content on the critical pore diameter of low-calcium high-strength cement pastes. Notably, when the fly ash content exceeds 10%, the critical pore diameter decreases at all ages, except for a slight increase at 3 d with 10% fly ash. At 1 d and 7 d, the critical pore diameter gradually increases with higher fly ash content, with a more significant effect observed at 1 d. Conversely, at 7 d, the critical pore diameter decreases with increasing fly ash content. At 28 d, the critical pore diameter remains unaffected by variations in fly ash content. 

(2) Ground limestone

**Figure 14:**
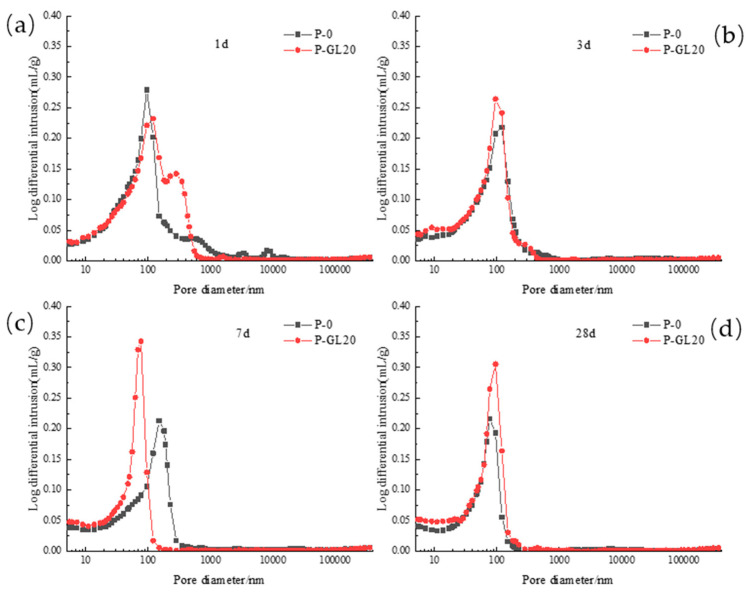
Differential curve of pore diameter distribution of low-calcium high-strength cement pastes with different dosages of ground limestone: (**a**) 1 d; (**b**) 3 d; (**c**) 7 d; and (**d**) 28 d.

Figure 14 illustrates the impact of ground limestone on the pore diameter distribution curve of low-calcium high-strength cement pastes at varying hydration ages. In the initial 24 h (1 d), both the P-0 and P-GL20 groups exhibit a peak and a rounded peak. However, the inclusion of ground limestone shifts the rounded peak towards smaller pore diameters and the primary peak towards larger pore diameters. During the curing period of 3 d to 7 d, both groups exhibit a single peak, with the addition of ground limestone causing the peak to shift towards smaller pore diameters and increase in intensity. This indicates that the incorporation of ground limestone facilitates the transition from macropores to smaller pores. At 28 d, both groups maintain a single peak, with ground limestone having minimal effect on the peak position but significantly enhancing the peak intensity. This suggests that the addition of ground limestone results in a more concentrated pore structure distribution. 

**Figure 15:**
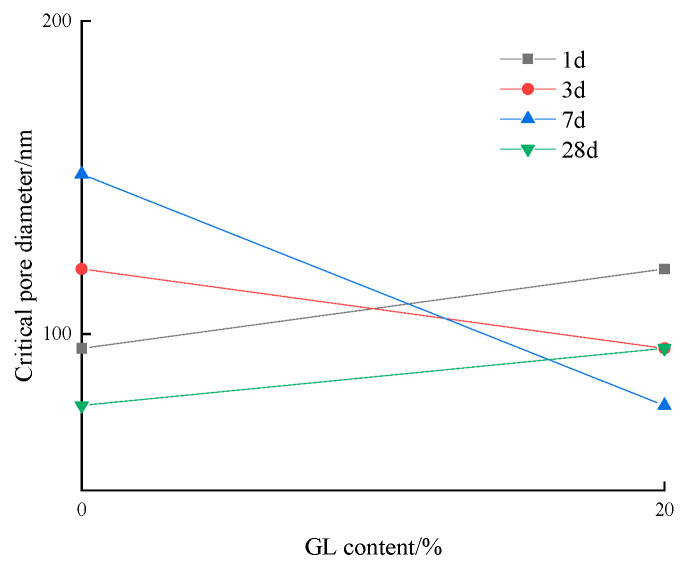
Effect of ground limestone content on the critical pore diameter of low-calcium high-strength cement pastes.

Figure 15 depicts the influence of ground limestone content on the critical pore diameter of low-calcium high-strength cement pastes. At the early age of 1 d and at 28 d, an increase in ground limestone content leads to a corresponding enlargement in the critical pore diameter of the cement pastes. Conversely, during the intermediate curing stages of 3 d and 7 d, an increase in ground limestone content results in a reduction in the critical pore diameter of the low-calcium high-strength cement pastes. 

(3) Slag

**Figure 16:**
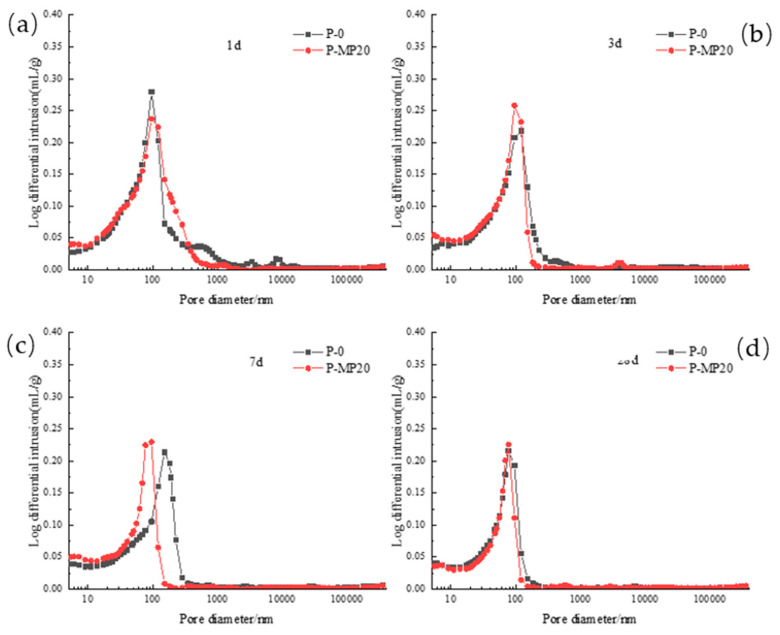
Differential curve of pore diameter distribution of low-calcium high-strength cement pastes with different dosages of slag: (**a**) 1 d; (**b**) 3 d; (**c**) 7 d; and (**d**) 28 d.

Figure 16 demonstrates the impact of slag on the pore diameter distribution differential curve of low-calcium high-strength cement pastes across various hydration ages. At 1 d, both the P-0 and P-MP20 groups exhibit a distinct sharp peak and a rounded peak. The inclusion of slag shifts the rounded peak towards larger pore diameters while reducing the intensity of both the rounded and sharp peaks. At the curing ages of 3 d, 7 d, and 28 d, both groups exhibit a single sharp peak. The addition of slag directs this peak towards smaller pore diameters, significantly increasing its peak intensity. This suggests that the incorporation of slag facilitates the transition from larger pores to smaller pores, with the most significant effect observed at 7 d. 

**Figure 17:**
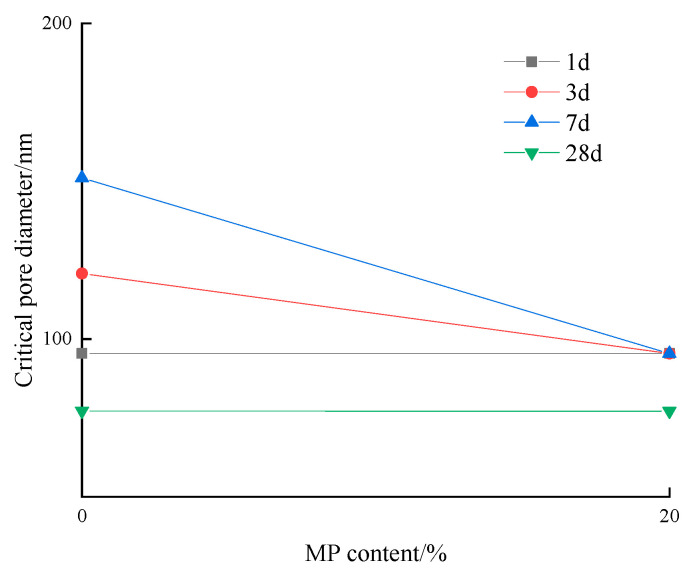
Effect of slag content on the critical pore diameter of low-calcium high-strength cement pastes.

Figure 17 shows the effect of slag content on the critical pore diameter of low-calcium high-strength cement pastes. The critical pore diameter of low-calcium high-strength cement pastes decreased with the increase in slag content at the age of 1 d~7 d, while the critical pore diameter of low-calcium high-strength cement pastes remained unchanged with the increase in slag content at 28 d age. 

## 4. Conclusions

(1) Under the condition of the same chloride ion concentration, the chloride binding capacity of the low-calcium high-strength pastes is stronger than that of the Portland cement pastes, because more Ms and C-S-H gel are formed in the later stage of the low-calcium high-strength cement. The use of fly ash and slag can improve the chloride binding capacity of low-calcium high-strength cement pastes, and if the content of ground limestone is greater than 10%, the chloride binding capacity of low-calcium high-strength cement pastes will be reduced, and the bound chloride of composite cement pastes will first increase and then decrease with the increase in ground limestone content. The addition of fly ash and slag can improve the chloride binding capacity of low-calcium high-strength cement pastes more effectively, and the effect of fly ash is better. The amount of bound chloride of low-calcium high-strength cement pastes and Portland cement pastes decreases with the increase in SO_4_^2−^ concentration, and the amount of bound chloride of Portland cement pastes is less affected by SO_4_^2−^ than that of low calcium high strength cement pastes.

(2) The incorporation of fly and ground limestone increases the porosity of low-calcium high-strength cement pastes, and increases with the addition of slag, while the addition of slag reduces the porosity of low-calcium high-strength cement pastes. Fly ash and ground limestone coarsen the pore structure of low-calcium high-strength cement pastes in the early stage and promote the transformation from large and medium pores to small pores in the later stage of hydration, thus optimizing the pore structure. The addition of slag has little effect on the pore structure of the low-calcium high-strength cement pastes in the early stage but promotes the transformation from large capillary pores to medium capillary pores in the later stage of hydration, and optimizes the pore structure. When the content of fly ash is more than 10%, the addition of fly ash reduces the critical pore diameter of low-calcium high-strength cement pastes at all ages, while the addition of 10% fly ash increases the 3 d critical pore diameter. The addition of ground limestone increases the critical pore diameter of low-calcium high-strength cement pastes and increases with the addition of ground limestone. In the early stage of hydration, the critical pore diameter of low-calcium high-strength cement pastes decreased with the increase in slag content, but in the later stage of hydration, the critical pore diameter remained unchanged with the increase in slag content.

## Figures and Tables

**Table 1:** Chemical composition of raw materials (wt, %).

Chemicals	SiO_2_	Al_2_O_3_	Fe_2_O_3_	CaO	MgO	SO_3_	Loss
LC	19.10	8.13	3.82	63.66	1.18	2.64	1.47
C	21.91	5.53	4.41	65.77	0.81	0.15	1.42
FA	49.4	39.81	1.86	4.17	0.78	0.65	3.33
GL	0.81	0.14	0.03	98.22	0.47	0.01	0.32
MP	32.20	17.84	0.30	37.94	7.81	1.56	2.35

**Table 2:** Mineral composition of raw materials (wt, %).

Minerals	C_3_S	C_2_S	C_3_A	C_4_AF	C_4_A_3_$	C$	C_12_A_7_	C_2_AS	C$H_2_
LC	41.58	24.14	6.05	10.62	4.83	9.00	0.22	0.23	
C	50.1	26.55	7.29	12.05					4

**Table 3:** Mix ratio of specimens.

Sample	LC	C	FA	GL	MP	W/C
P-0	100	0	0	0	0	0.4
P-1	0	100	0	0	0	0.4
P-FA10	90	0	10	0	0	0.4
P-FA20	80	0	20	0	0	0.4
P-FA30	70	0	30	0	0	0.4
P-GL10	90	0	0	10	0	0.4
P-GL20	80	0	0	20	0	0.4
P-GL30	70	0	0	30	0	0.4
P-MP10	90	0	0	0	10	0.4
P-MP20	80	0	0	0	20	0.4
P-MP30	70	0	0	0	30	0.4

**Table 4:** Composition of chloride solutions.

		mol/L
No.	NaCl	NaSO_4_
1	0.5128	0
2	0.5128	0.1408
3	0.5128	0.2816
4	0.5128	0.4224

**Table 5:** Amount of bound chloride in hydrated low-calcium high-strength cement pastes and Portland cement pastes of chloride solutions after equilibrium. (Error: 2.2–2.9%).

No.	C_b_(mg/g)
P-0	4.70
P-1	4.25

## Data Availability

Data are contained within the article.
